# Regulation of Myelination in the Central Nervous System by Nuclear Lamin B1 and Non-coding RNAs

**DOI:** 10.1186/2047-9158-3-4

**Published:** 2014-02-05

**Authors:** Shu-Ting Lin, Mary Y Heng, Louis J Ptáček, Ying-Hui Fu

**Affiliations:** 1 Department of Neurology, University of California, 1550 Fourth street, UCSF-Mission Bay, Rock Hall 548, San Francisco, CA 94158, USA; 2 Howard Hughes Medical Institute, University of California, San Francisco, CA 94158, USA

**Keywords:** Lamin, Long non-coding RNA, MicroRNA, Myelin

## Abstract

Adult-onset autosomal dominant leukodystrophy (ADLD) is a progressive and fatal hereditary demyelination disorder characterized initially by autonomic dysfunction and loss of myelin in the central nervous system (CNS). Majority of ADLD is caused by a genomic duplication of the nuclear lamin B1 gene (*LMNB1*) encoding lamin B1 protein, resulting in increased gene dosage in brain tissue. *In vitro*, excessive lamin B1 at the cellular level reduces transcription of myelin genes, leading to premature arrest of oligodendrocyte differentiation. Murine models of ADLD overexpressing *LMNB1* exhibited age-dependent motor deficits and myelin defects, which are associated with reduced occupancy of the Yin Yang 1 transcription factor at the promoter region of the proteolipid protein gene. Lamin B1 overexpression mediates oligodendrocyte cell-autonomous neuropathology in ADLD and suggests lamin B1 as an important regulator of myelin formation and maintenance during aging. Identification of microRNA-23 (*miR-23*) as a negative regulator of lamin B1 can ameliorate the consequences of excessive lamin B1 at the cellular level. *miR-23a*-overexpressing mice display enhanced oligodendrocyte differentiation and myelin synthesis. *miR-23a* targets include a protein coding transcript *PTEN* (phosphatase and tensin homolog on chromosome 10), and a long noncoding RNA (*2700046G09Rik*), indicating a unique role for *miR-23a* in the coordination of proteins and noncoding RNAs in generating and maintaining healthy myelin. Here, we provide a concise review of the current literature on clinical presentations of ADLD and how lamin B1 affects myelination and other developmental processes. Moreover, we address the emerging role of non-coding RNAs (ncRNAs) in modulating gene networks, specifically investigating *miR-23* as a potential target for the treatment of ADLD and other demyelinating disorders.

## Introduction

Compact concentric wraps of myelin, a unique characteristic of vertebrates, can speed up propagation of electrical activities along axons in the nervous system. Myelin sheath consists of membranous outgrowth from specified glia such as oligodendrocytes in the central nervous system (CNS) and Schwann cells in the peripheral nervous system. In adults, oligodendrocytes are generated by proliferation or migration of oligodendrocyte progenitors cells (OPC) throughout the CNS, followed by differentiation into post-mitotic pre-myelinating oligodendrocytes and myelination of adjacent axons when appropriate environmental cues are present [1-6]. Molecular mechanisms underlying oligodendrocyte differentiation and CNS myelination include intrinsic cues such as transcriptional factors, microRNAs (miRNAs), and chromatin remodeling, and extrinsic signaling such as extracellular ligands and neuronal activities [1]. A number of transcription factors have been identified that promote oligodendrocyte specification [7,8], differentiation, myelination [7-13], or prevent OPC from differentiating [14-17]. Failure to integrate these molecular mechanisms may lead to myelin malformation or premature breakdown of myelin similar to that observed in human diseases such as hereditary leukodystrophies or multiple sclerosis. In this review, we address adult onset autosomal dominant leukodystrophy (ADLD), recently shown to be caused by increased expression of lamin B1 resulting from duplication of the gene encoding lamin B1, *LMNB1*[18-24], or possibly its dysregulation [25]. Importantly, the adverse effect of *LMNB1* overexpression in oligodendrocytes can be abrogated by *miR-23*, suggesting that it may be a negative regulator of lamin B1 [26]. Therefore, we will discuss the clinical presentation of ADLD, and how lamin B1 affects myelination (both when in excess and in its absence). Moreover, we discuss the emerging role of non-coding RNAs (ncRNAs) in modulating gene networks, specifically investigating *miR-23* as a potential target for the treatment of ADLD and other demyelinating disorders.

## Clinical presentation of adult-onset autosomal dominant leukodystrophy

ADLD was originally reported for a large American-Irish family carrying a progressive and fatal neurological white matter disorder [27]. Additional ADLD families were subsequently found in other ethnic groups [19,28-33]. Typical ADLD symptoms begin between the forth and sixth decades of life with early presentation of autonomic symptoms, including bowel/bladder dysfunction, impotence in males, and orthostatic hypotension [34-36]. Recent evidence of delayed onset of autonomic dysfunction in a Serbian family with *LMNB1*-dupication presents a variant of the ADLD symptoms [31]. Clinically, it is a progressive degenerative neurological disorder characterized by pyramidal, cerebellar, and autonomic abnormalities [27,29,30,37]. Autonomic symptoms are followed by cerebellar impairment (ataxia, dysmetria, nystagmus, and action tremors) and pyramidal abnormalities (spasticity and weakness of both upper and lower extremities). Cardiovascular and pharmacological assessments indicate a selective sympathetic failure with spared cardiovagal response [36,38]. Mild cognitive, visual, and auditory abnormalities are also found in some cases. Selective loss of noradrenergic fibers with preserved cholinergic fibers in skin biopsy of a patient with ADLD [36] indicates autonomic neuropathy. However, peripheral nerve, sympathetic ganglia, vagus nerve, and adrenal medulla did not exhibit obvious abnormality at autopsy [38]. The MRI scans display diffused, confluent and symmetric white matter degeneration starting in the frontoparietal region, extending towards the brainstem and cerebellar white matter [30,35,39]. Periventricular white matter appears less involved than the adjacent white matter [30]. The diameter of the medulla oblongata is reduced in the coronal plane and the corpus callosum is also atrophic. White matter changes are most significant in the brain though spinal cord involvement has been reported in some cases [40]. Neuropathological findings reveal white matter abnormalities in the frontoparietal and cerebellar white matter but spare the cortex and subcortical U fiber in symptomatic ADLD patients [30,35]. Light microscopy showed vacuolated white matter with no significant loss of oligodendroglia, which has no clear sign of inflammatory changes and reactive astrocytosis [30,35]. In addition, sparse astrocytes with intense immunoreactivities against insulin-like growth factor-1 and vimentin are accompanied by beaded/foreshortened morphology or thickening processes. Reduced numbers of Purkinje cells and slightly increased numbers of Bergmann cells in cerebellum are also found [30]. Lacking significant inflammatory infiltrates, activated microglia, or macrophages, this disorder does not appear to result from the direct autoimmune attack on myelin proteins that is present in multiple sclerosis. Overall, preservation of oligodendroglia in the presence of demyelination and the lack of or moderate astrogliosis under light microscopy are the unique features of ADLD [30,35]. Genetic evidence demonstrates that ADLD is caused by duplication of *LMNB1* in the majority of ADLD patients [18-24], and recent study from twenty families revealed a minimal 72 kb of *LMNB1* duplication required for the disease [23]. However, how this mutation leads to these described cellular phenotypes is not completely clear. Therefore, in the next section we survey current research on lamin B1 and how over- or under-expression of this protein can dysregulate myelination.

## Lamin B1 is integral to the nuclear lamina and regulates gene expression

Lamins are structural components of the nuclear lamina, which is a filamentous meshwork of proteins underlying the inner nuclear membrane. Nuclear lamina has been found to play dynamic roles in the organization and regulation of chromatin, transcription, DNA replication, DNA repair, and various epigenetic phenomena such as euchromatin and heterochromatin transitions [41]. RNA interference-mediated knocking down of the only lamin, Ce-lamin, in *C. elegans* leads to embryonic lethality due to defects in cell cycle progression, chromosome segregation, chromatin organization and correct spacing of nuclear pore complexes [42]. Similarly, depletion of the B-type lamin, lamin Dm0, in *Drosophila* cultured cells and embryos by RNA interference results in morphological alterations of nuclei, nuclear fragility and the arrest of embryonic development [43]. Mammalian cells have two major types of lamins, A-type and B-type, and mutations in genes encoding the nuclear lamins can cause a wide range of human diseases, collectively called laminopathies (for review, see [44-47]). B-type lamins include lamin B1 encoded by *LMNB1* (mouse *Lmnb1*) and lamin B2 encoded by *LMNB2* (mouse *Lmnb2*). Lamins anchor chromatin to the nuclear lamina and act as a scaffold for chromatin remodeling, and are thus critical for determining spatial organization of chromosomes in the nucleus [48]. Furthermore, genomic regions tethered to the nuclear periphery were shown to be associated with reduced transcription of genes in the region, suggesting that lamins may play an active role in regulating transcription as well [49,50]. Cells isolated from human with laminopathies or murine models with lamin deficiency all displayed abnormal nuclear structures [51-53]. Numerous diseases are associated with *LMNA* mutations, with symptoms ranging from myopathy, lipodystrophy, accelerated aging disorders, peripheral neuropathy, to bone disorders [46,54]. Recent studies in murine models of *Lmna* mutations suggest that mutations in the A-type lamin confer phenotypes by gain-of-function toxicity for some models and loss-of-function for others [46]. In contrast, myelin disease is the only reported phenotype associated with *LMNB1* duplications [18] while acquired partial lipodystrophy is associated with mutations in *LMNB2*[55]. Lamin B1 has been reported to play a role in cell proliferation and senescence in culture [56-58], and both silencing and overexpressing lamin B1 have been linked to senescence [56,57,59]. However, the B-type lamins do not affect the development of skin keratinocytes and hepatocytes [60,61], which appear morphologically normal in histological analyses. Given that lamin B1 binds to genes whose expression are not essential for proliferation and survival or *in vitro* lineage specification in embryonic stem cells [62], we propose that lamin A and lamin B1 control different sets of gene expression through direct chromatin-binding, and that B-type lamins may specifically affect neural development, whereas A-type lamins may preferentially affect other cell types. During differentiation, differences in temporal and spatial expression of A-type and B-type lamins will result in different chromatin patterns, thus conferring unique transcriptome signatures of cell lineage.

## Lamin B1 is necessary for proper cellular development

Consistent with its proposed role in regulating specific gene expression, lamina-associated domains of lamin B1 are connected to genome regions with low expression levels, exhibiting lower levels of active chromatin, and enriched with silenced chromatin markers that indicate repressive chromatin organization [62-65]. The high-resolution genome-nuclear lamina interaction maps of lamin B1 in pluripotent embryonic stem cells, multipotent precursor cells, and terminal differentiated cells revealed a dynamic interaction of nuclear lamina and genes in the genome according to cell type, differentiation steps, and gene expression levels that correlate with subsequent repression or activation [66]. These unique characteristics of lamin B1 suggest its importance in cellular development that requires temporal and spatial regulation of gene networks.

Supporting this hypothesis, *Drosophila* testis’ nuclear lamin-B regulates cyst stem cell differentiation and organization of the niche through nuclear epidermal growth factor receptor signaling [67]. Furthermore, deficiencies of lamin B1 and/or lamin B2 in mice lead to perinatal lethality, especially due to poor lung development [51,62,68,69]. Knockout of *Lmnb1/2* or *Lmnb1* alone also results in microcephaly. Despite developmental impairment, lamin B2 deficiency has comparatively better-developed lung, brain, and diaphragm, suggesting a possible functional redundancy between the B-type lamins [62,68]. In recent years, lamin B1 and lamin B2 have been demonstrated to play a critical role in neuronal migration in mouse cerebral cortex and cerebellum [52,62,68]. Impairment of neuronal migration in cortex was attributed to improper connection between cytoskeleton and the nucleus [62], which is consistent with increased nuclear spinning (an indication for impairment in anchoring the nucleus to cytoskeleton) in fibroblasts cultured from *Lmnb1*^Δ/Δ^ mice [70]. Similarly, spindle orientation defects have been identified in cerebral cortex in *Lmnb1* knockout mice that lead to defects in division of neural progenitor cells [71]. In addition, accelerated cell cycle exit and enhanced apoptosis in cerebral cortex of *Lmnb1* knockout mice suggest that lamin B1 may also modulate proliferation potential and differentiation of neural progenitor cells and neurons in the embryonic brain [62].

The mammalian lamin B1 is post-translationally modified by farnesylation, endoproteolysis, and carboxymethylation at a carboxyl-terminal CaaX motif [72], and its specific interacting portions appear to be important for its function. For example, gene trap insertion in mouse resulting in an allele encoding lamin B1 lacking the C terminus causes perinatal lethality due to lung and bone abnormalities [51]. Furthermore, impaired farnesylation of lamin B1 leads to loss of chromatin anchor to the nuclear lamina, and causes migratory defects of neurons in brains and perinatal lethality [73]. Together, these results suggest that developmental processes are highly sensitive to both the expression level and post-translational modifications of lamin B1. Interestingly, lamin B1 itself in the brain is developmentally regulated, with levels peaking at birth or postnatal day 1, followed by a gradual decrease from postnatal day 1 to 10 months of age [26] whereas levels of many myelin proteins in the murine brain gradually increase with age in a pattern that is complementary to that of lamin B1. These results imply a possible role for lamin B1 in the regulation of oligodendrocyte development or myelin proteins expression.

## Lamin B1 overexpression leads to demyelination

Overexpression of the B-type lamins was previously shown to promote nuclear membrane growth and intranuclear membrane formation in amphibian oocytes, epithelium, and mammalian kidney cells in a CaaX motif-dependent manner [74,75]. In agreement with these findings, ectopic overexpression of lamin B1 in neural and glial cell lines increase surface area of nuclear membrane and intranuclear aggregates [26]. Increased expression of lamin B1 also results in perturbations of inner nuclear membrane proteins, chromatin organization, and nuclear pore transport. It is possible that formation of abnormal intranuclear membranes caused by excessive lamin B1 production leads to the altered subcellular localization of nuclear envelope proteins and perturbed nuclear transport, especially in oligodendrocytes. Supporting this hypothesis, widespread overexpression of lamin B1 in mouse brain using bacterial artificial chromosome (BAC) transgenic engineering recapitulates many of the clinical features present in ADLD, including aberrant myelin formation, axonal degeneration and demyelination [76]. *LMNB1*^BAC^ mice exhibit seizure, cognitive impairment, and motor deficits, which progressively worsen with age. Remarkably, lineage-specific overexpression of lamin B1 in mouse oligodendroglia is sufficient for reproducing the histopathological, molecular and behavioral abnormalities in ADLD. Although decreased abundance of myelin protein, proteolipid protein (PLP), attributed to loss of occupancy of a transcription activator, Yin Yang 1 (YY1) on its promoter, contributes at least in part to the myelin phenotype in ADLD, the possibility that an independent degenerative process of myelin also contributes to the phenotype has not been excluded. Nevertheless, these results suggest that genes required for myelin proteins and oligodendrocyte maturation are sensitive to changes in lamin B1 abundance. Indeed, the effects of lamin B1 overexpression on gene transcription were demonstrated by the repression of myelin-specific genes [76] and the activation of GFAP transcription in oligodendroglial cell lines [26]. Lamin B1 is associated with an oxidative stress pathway transcriptional factor, octamer transcription factor 1 (Oct-1), in normal mouse fibroblasts [77]. Fibroblast cells lacking full-length lamin B1 are vulnerable to oxidative stress similar to what is found in Oct-1 knockout cells. In fibroblasts cultured from ADLD patients, Oct-1 was increased at the nuclear periphery with reduced nucleoplasmic localization under oxidative stress conditions [78]. However, whether Oct-1 participates in the regulation of myelin gene expression and other developmental defects in the CNS require further investigation. Supporting *in vivo* findings, in a primary culture system, transient overexpression of lamin B1 in oligodendrocytes by lentiviral transduction causes defects in myelin protein expression and differentiation accompanied by mild nuclear envelope growth/distortion [26]. Overall, these results indicate that overexpression of lamin B1 could disturb the unique gene expression patterns in individual CNS cell types and that these phenotypes would only appear in certain cell types that are vulnerable to transcriptional perturbance during differentiation. These studies identify a mechanism by which excessive lamin B1 expression can cause oligodendrocyte cell-autonomous neuropathology in ADLD, and implicate lamin B1 as an important factor for myelin formation and maintenance. Therefore, identifying upstream regulators of lamin B1 may provide a means of treating de/dysmyelinating disorders such as ADLD.

## *MiRNA-23* regulates myelination through multiple targets including *LMNB1*

Recently, miRNAs are implicated to regulate a large number of developmental processes and diseases through fine-tuning biological networks [79-84]. Expression levels of miRNAs in oligodendroglia vary according to their state of differentiation, indicating a possible role for miRNAs in regulating oligodendrocyte development [85-88]. Notably, specific miRNAs regulate various important factors in progenitor stages and during OPC differentiation [87-89]. In addition, ablation of miRNA cluster (*miR-17-92*) perturbs oligodendrocyte proliferation through targeting phosphatase and tensin homolog on chromosome 10 (PTEN) [86]. Moreover, disruption of miRNA biogenesis by Dicer ablation in oligodendroglia at post-developmental stages results in a neurodegenerative phenotype including demyelination, inflammation, and axon loss [90], suggesting that miRNAs are also important for myelin maintenance at later developmental stages. Therefore, expression of miRNAs at different stages is thought to promote differentiation by antagonizing suppressive factors in progenitors and/or to keep oligodendrocytes at a mature stage for myelin maintenance.

As excessive lamin B1 expression appears to be the sole cause of ADLD, understanding its regulation may provide insight into potential treatment methods. To determine whether lamin B1 can be regulated by miRNAs, several miRNAs predicted to target *LMNB1* were screened (TargetScan: http://www.targetscan.org/vert_42/). *miRNA-23* (*miR-23*) is among the most abundant miRNAs in oligodendrocytes [85,86] and is able to counteract the expression of *Lmnb1*[26,57]. In the presence of excess *miR-23* in cell culture, a greater proportion of cells express mature markers of oligodendrocytes that are accompanied by multipolar morphological appearance and increased levels of mature myelin proteins, indicating that *miR-23* can enhance differentiation. In contrast, excessive lamin B1 leads to defective differentiation of oligodendrocytes. Importantly, the adverse effect of lamin B1 on oligodendrocyte cells can be abrogated by *miR-23* as a negative regulator of lamin B1 [26]. Consistent with these observations, the developmental expression pattern of *miR-23 in vivo* is reciprocal to that of the lamin B1 [26,91].

**Figure 1:**
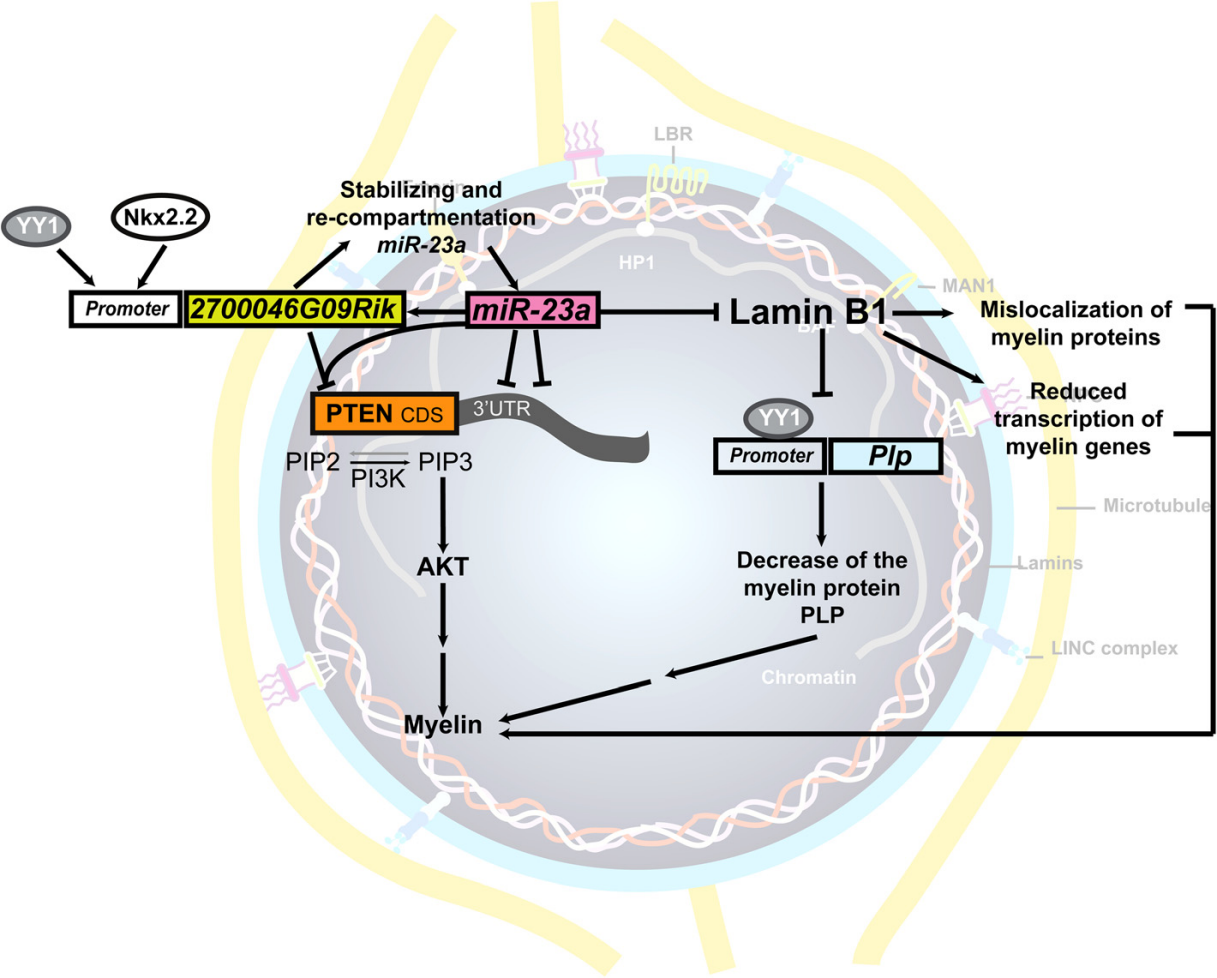
**Model for the mechanism of *miR-23a *in regulating myelination.***MiR-23a* enhances *2700046G09Rik* and represses PTEN and lamin B1 in oligodendroglia. Lamin B1 overexpression reduces the occupancy of YY1 on *Plp* promoter, decreases transcription of myelin genes, and causes mislocalization of myelin proteins. *2700046G09Rik* interacts with and stabilizes *miR-23a.* In addition, *2700046G09Rik* re-compartments and alters *miR-23a* sub-cellular localization, which further enhances repressive effects of *miR-23a* on PTEN. Less PTEN in antagonizing PI3K activity leads to AKT activation which then results in myelination. *miR-23a* represses lamin B1 leading to increased myelination.

The *in vivo* effects of *miR-23* on oligodendrocyte differentiation and myelin formation in the CNS were validated by transgenic mice overexpressing *mmu-miR-23a* driven by an oligodendrocyte specific promoter [2’ , 3’-cyclic ucleotide 3’-phophodiesterase (*Cnp*)] [91]. These mice exhibit increased myelin thickness, providing *in vivo* evidence that *miR-23a* enhances myelin synthesis [91]. To explore possible *miR-23a* targets that are important for CNS myelination, RNA-Seq approach was applied to examine oligodendrocytes derived from both *miR-23a* transgenic mice and control littermates. PTEN was identified as a potential target and further characterization confirmed that the PTEN/PI3K/Akt/mTOR pathway is modulated by *miR-23a* (Figure 1). Interestingly, a long non-coding RNA (lncRNA) neighboring PTEN, *2700046G09Rik*, was identified in the same study as another *miR-23a* target and modulates PTEN itself in a *miR-23a* dependent manner. These data implicate a novel role for *miR-23a* in the coordination of proteins and non-coding RNAs for generating and maintaining healthy myelin [91]. lncRNAs have recently been shown to function as transcriptional and post-transcriptional regulators of gene expression, such as through modulation of chromatin modifications, or transcriptional interference by antisense transcription [83,92]. *miR-23a* upregulates *2700046G09Rik* transcription, and *2700046G09Rik* in turn lengthens the half-life of *miR-23a*, thus potentiating its repressive effects. *2700046G09Rik* can also target its neighboring gene *PTEN* and lead to a reduction in PTEN levels. Therefore, repressive effects on PTEN can either occur with *miR-23a* alone or in coordination with *2700046G09Rik*. In addition, *2700046G09Rik* may aid in the cellular re-compartmentation of *miR-23a* into P-bodies, which could also contribute to the regulation of PTEN levels, suggesting that interplay of *miR-23a* and *2700046G09Rik* infers additional molecular regulation of mRNA decay [91].

*2700046G09Rik* is expressed higher in oligodendrocytes under differentiation conditions than OPCs or astrocytes. The presence of *2700046G09Rik* in oligodendroglia potentiates and signals the activation of the *miR-23a*-PTEN/Akt-mTOR cascades in the correct developmental stage, thus regulating the expression of myelin genes in oligodendrocytes. In addition, two important transcription factors (YY1 and Nkx2.2) for oligodendroglia [10,11,93] act on the promoter of *2700046G09Rik*, leading to its transactivation during oligodendrocyte development. These findings are consistent with the emerging notion that dynamic changes of lncRNAs are important for glia differentiation [94]. Intriguingly, YY1 occupancy at PLP promoter is reduced in the *LMNB1*^BAC^ mice as described earlier [76]. All in all, these findings further highlight the complexity of oligodendrocyte/myelin regulatory pathways, as demonstrated by the coordination of transcriptional (transcription factors) and post-transcriptional (miRNA and lncRNA) mechanisms.

## Conclusion

Taken together, *in vitro* and *in vivo* investigations of ADLD have established the importance of nuclear lamins, miRNA, and ncRNA in oligodendroglial development, and provide potential therapeutic approaches for myelin diseases. For example, *miR-23a* may ameliorate reduced levels of oligodendrocyte- and myelin-specific proteins in ADLD. In addition, *miR-23* can also enhance oligodendrocyte development through other lamin B1 independent pathways such as PTEN/Akt/mTOR. Genetic interaction studies using mouse models in the future will further reveal lamin B1 dependent and independent effects of *miR-23a*, and thus determine the possibility of developing non-coding RNAs as potential therapeutic intervention as well. Although ADLD is a rare disease, it has provided us with new knowledge on nuclear events underlying myelin maintenance and events leading to premature myelin breakdown [76]. Understanding the function of lamins in the context of lamin-binding-chromatin organization will also unveil novel mechanisms that mediate myelin diseases, aging process, and other biological functions.

To date, the duplication of *LMNB1* is the only disease that links nuclear structure to myelin formation in the CNS [18]. Interestingly, duplication of *PLP*1 is one of the most common causes of demyelination in Pelizaeus-Merzbacher disease [95,96]. Overexpression of the common myelin protein, PLP, causes endoplasmic reticulum stress, leading to oligodendrocyte demise [97]. It will be intriguing to investigate threshold effect of gene transcription and translation on myelin formation and other biological mechanisms where gene and protein expression homeostasis are critical. Likewise, identification of upstream enhancer/repressor elements that can modulate miRNA expression and additional downstream effectors of lamin B1 and *miR-23* may provide novel insights into the mechanisms of oligodendrocyte development, myelin formation, and maintenance. The *LMNB1*^BAC^ mouse model overexpressing lamin B1, mimicking human ADLD, will be a critical resource for further investigations *in vivo*, because while increased lamin B1 expression in neurons or glia causes lethality in *Drosophila*[18], the striking similarities between human and murine oligodendrocytes may hasten investigations toward clinical development. Growing understanding of human-relevant pathways critical for myelin regulation will provide novel screening targets for the treatment of ADLD and other myelin-based disorders.

## Competing interests

The authors declare that they have no competing interests.

## Authors’ contributions

STL and MYH carried out literature search and drafted the manuscript. LJP and YHF, the supervisors of the research group, provided the guidance and instructions and critically revised the manuscript. All authors read and approved the final manuscript.
